# Are men under-treated and women over-treated with antidepressants? Findings from a cross-sectional survey in Sweden

**DOI:** 10.1192/pb.bp.116.054270

**Published:** 2017-06

**Authors:** Lena Thunander Sundbom, Kerstin Bingefors, Kerstin Hedborg, Dag Isacson

**Affiliations:** 1 Uppsala University, Uppsala, Sweden; 2 University of Gävle, Gävle, Sweden

## Abstract

**Aims and method** To examine gender differences in self-reported depression and prescribed antidepressants (ADs). The Hospital Anxiety and Depression Scale was used to assess depression, and information on prescribed ADs was obtained from the Swedish Prescribed Drug Register.

**Results** Depression was reported by 11.7% of the participants (12.3% men and 11.2% women). ADs were prescribed for 7.6% of the participants (5.3% men, 9.8% women). Among men, 1.8% reported depression and used ADs, 10.5% reported depression but did not use ADs, and 3.6% used ADs but did not report depression. The corresponding figures for women were 2.6%, 8.6% and 7.2%.

**Clinical implications** Men report depression to a greater extent than women but are prescribed ADs to a lesser extent, possibly a sign of under-treatment. Women are prescribed ADs without reporting depression more often than men, possibly a sign of over-treatment. Although the causes remain unclear, diagnostic and treatment guidelines should benefit from considering gender differences in these respects.

Depression is currently considered one of the largest and fastest growing health hazards.^1^ Although only a small percentage of all those with mental health problems contact healthcare professionals and obtain a diagnosis, depression is one of the most common causes of sick leave and disability.^2–5^ Diagnosed depression was relatively unusual 20 years ago, but the incidence has increased remarkably with the introduction of new diagnostic guidelines and antidepressant drugs. What was previously considered psychological distress was then interpreted as a disease, with the risk of over-diagnosis and over-treatment.^6–10^ The point prevalence of depression in the general population is now estimated as 3–9%.^1,11,12^ There is an explicit gender impact on diagnosed depression, with a 2:1 ratio of women/men; about one in four women and one in ten men will develop depression severe enough to require treatment at some time in their lives.^1,2,11,12^ Moreover, depression may present differently in women and men. Women may be more prone to somatic symptoms of depression, whereas men appear to have more melancholic symptoms and to be more susceptible to drug misuse and aggressive behaviour.^13–17^ To date, however, there is no clear understanding of what causes these gender disparities in depression. They are considered likely to be a combination of several factors: biological, social and behavioural.^18,19^

Depression is a long-lasting and, if left untreated, often chronic condition. Treatment usually lasts at least 6–12 months, and includes pharmacological therapy with antidepressants (ADs).^20^ The use of ADs has increased dramatically in recent years.^21^ According to the national Swedish Prescribed Drug Register (SPDR), almost 9% of the Swedish population was prescribed ADs in 2014, and 65% of these ADs were prescribed for women.^22^ Similar patterns have been found in other countries.^23–25^ The explanation for this escalation, especially seen in women, remains unclear, but has sometimes been interpreted as a sign of inappropriate use.^8,9^ Nevertheless, despite the widespread use of ADs, depression has repeatedly been shown to be inadequately treated in the general population. Some studies have found that fewer than one in four patients with depression are prescribed ADs and that the duration of treatment is often shorter than recommended.^26,27^

The ADs prescribed are primarily selective serotonin reuptake inhibitors (SSRIs), although others, for example serotonin–noradrenaline reuptake inhibitors (SNRIs), tricyclic antidepressants (TCAs) and monoamine oxidase inhibitors (MAOIs), are also used depending on illness severity, the patient's age and various adverse drug reactions.^20,28,29^ Gender impact has been observed not only on the number of ADs prescribed but also on their type. For example, women are prescribed SSRIs more often than men.^30^

We examined gender differences in the relationship between self-reported depression and prescribed ADs, in the prevalence of self-reported depression, and in the number and type of prescribed ADs.

## Method

### Participants

**Fig 1:**
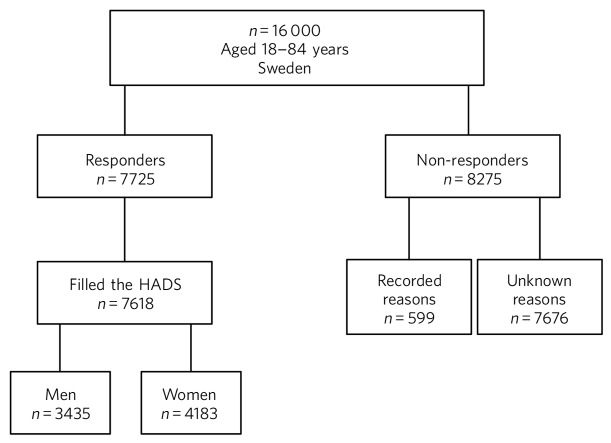
Study population, responders and non-responders, Sweden 2012/2013.

A questionnaire was sent to a random sample (*n* = 16 000, aged 18–84 years) of the Swedish population (totaling 9.5 million); responses were received from 7725 people (48.3%), as presented in Fig. 1. The study complies with ethical research requirements, as approved by the Regional Ethical Review Board in Uppsala, Sweden (Dnr 2012/073). Participation in the study was voluntary and information about its purpose was sent out with the questionnaire. Filling in and returning the questionnaire was considered to be equivalent to the respondent giving their agreement to participate in the study.

### Assessment of depression and prescribed antidepressants

Self-reported depression was assessed using the Hospital Anxiety and Depression Scale (HADS).^31^ Of the 7725 available participants, 7618 (3435 men, 4183 women) filled in the HADS form (Fig. 1) and all analyses were based only on these participants. The HADS was developed to detect patients with high levels of psychological distress and does not include assessment of somatic symptoms. It contains two subscales, one each for anxiety and depression, each consisting of 7 items (score range 0–21) capable of distinguishing between these diagnoses. Higher scores indicate higher levels of psychological distress. Each subscale has three categories based on the score: 0–7 (normal), 8–10 (borderline) and 11–21 (abnormal). In this study, a cut-off level of +8 was used on the depression scale; this level indicates at least mild depression and provides an optimal balance between sensitivity and specificity.^32^

Information on depression obtained from the HADS was linked (through the participants' identification number, a unique lifetime personal identifier given to all Swedish citizens) to prescription data. Prescribed ADs were obtained from the SPDR, a national register held by the National Board of Health and Welfare, which gathers data on all dispensed prescriptions for patients in ambulatory care from the entire Swedish population.^22,33^ We collected information on ADs 0–6 months prior to the HADS evaluation. SPDR drugs are classified according to the Anatomical Therapeutic Chemical (ATC) classification system.^34^ The ADs (N06A) were categorised as TCAs (N06AA, e.g. amitriptyline, imipramine), SSRIs (N06AB, e.g. citalopram, fluoxetine), ‘others’ (N06AX, including SNRIs (e.g. venlafaxine) and tetracyclic antidepressants (TeCAs, e.g. mirtazapine)), and monoamine oxidase inhibitors (MAOIs; N06AF, N06AG, e.g. moclobemide). The MAOIs were excluded due to few users.

### Analyses

The Statistical Analysis System software (SAS9.2, Cary, North Carolina, USA) was used to perform chi-squared tests (χ^2^, *P*) to examine gender differences in the relationship between self-reported depression and prescribed ADs, and in prevalence of self-reported depression, prescribed ADs, and type of ADs prescribed. Logistic regression analysis (odds ratios (OR) with 95% confidence intervals) was used to examine gender differences in self-reported depression, controlling for age.

## Results

In total, 11.7% of the study population (12.3% men, 11.2% women; χ^2^, n.s.) was classified as having self-reported depression. Logistic regression analysis showed that the difference between men and women was statistically significant, i.e. men reported depression more often than women (OR 1.226 (CI 1.062–1.414)). According to the SPDR, 7.6% of the study population had been prescribed at least one AD during the 6 months prior to the HADS evaluation. Significantly more women than men were prescribed ADs: 5.3% of the men and 9.8% of the women were prescribed at least one AD (*P*<0.0001).

Table 1 presents gender differences in the relation between prescribed ADs and self-reported depression in the study population. Among the men, 1.8% reported depression and used ADs, 10.5% reported depression but did not use ADs, and 3.6% used ADs but did not report current depression, while 84.1% were neither depressed nor used ADs. The corresponding figures for women were 2.6%, 8.6%, 7.2% and 81.6%. The gender difference was statistically significant (χ^2^ *P*<0.001) in all age groups except the youngest, and was most marked in the groups aged 45–64 and 65–74 years.

**Table 1:** Relation between self-reported depression (assessed using the HADS) and prescribed antidepressants (ADs) in the study population (*n* = 7618), Sweden 2012/2013

	Men					Women					
		Depression		No depression			Depression		No depression		
		AD-users	Non-users	AD-users	Non-users		AD-users	Non-users	AD-users	Non-users	

Age (years)	*n*		%			*n*			%		*P*^a^
18–34	593	1.7	8.9	2.0	87.4	831	1.8	9.8	3.6	84.8	N.S.

35–44	475	1.9	11.8	2.3	84.0	576	3.1	8.7	6.8	81.4	<0.01

45–64	1277	1.8	10.6	4.6	82.9	1537	3.3	9.0	8.3	79.4	<0.001

65–74	740	1.5	9.9	3.6	85.0	811	2.1	6.8	9.2	81.9	<0.001

75–84	350	2.3	12.6	3.7	81.4	428	1.9	7.9	7.0	83.2	<0.05

Total	3435	1.8	10.5	3.6	84.1	4183	2.6	8.6	7.2	81.6	<0.001

a. χ^2^ analyses comparing men and women.

The participants who had received at least one prescribed AD during the 6 months studied (*n* = 592: men *n* = 182, women *n* = 410) were analysed with respect to the type of AD prescribed (Table 2). SSRIs were the most commonly prescribed ADs for both men (62.8%) and women (71.0%), although women were prescribed them more often than men, particularly in the age group 45–64 years (χ^2^ *P*<0.05). By contrast, there was no statistically significant gender difference for the TCAs (men 14.8%, women 10.2%), except for in the age group 45–64 years. Further, men were prescribed ‘other’ ADs (e.g. SNRIs and TeCAs) significantly more often than women (men 39.3%, women 28.1%; χ^2^ *P*<0.01).

**Table 2:** Types of antidepressant (ATC classification) among participants prescribed at least one antidepressant (*n* = 592), by age and gender, Sweden 2012/2013

	Users, *n*			SSRIs (N06AB)			TCAs (N06AA)			Others (N06AX)^a^		
Age (years)	Total	Men	Women	Men	Women	*P*^b^	Men	Women	*P*^b^	Men	Women	*P*^b^
18–44	144	42	102	69.1	73.5	NS	9.5	5.9	NS	38.1	29.4	NS

45–64	260	82	178	58.5	71.3	<0.05	20.7	12.4	<0.05	37.8	25.8	<0.05

65–84	188	58	130	64.4	68.5	NS	10.2	10.8	NS	42.4	30.0	<0.05

Total	592	182	410	62.8	71.0	<0.05	14.8	10.2	NS	39.3	28.1	<0.01

ATC, Anatomical Therapeutic Chemical; NS, not significant; SSRIs, selective serotonin reuptake inhibitors; TCAs, tricyclic antidepressants.

a. For example, serotonin–noradrenaline reuptake inhibitors, tetracyclic antidepressants.

b. χ^2^ analyses comparing men and women.

## Discussion

The present study found that the relationship between self-reported depression and prescribed ADs differs by gender. As in several other studies,^26,27^ the majority of those who reported depression in our study did not use ADs, and overall, men used ADs to a lesser extent than did women, although they reported depression to a greater extent. This could have been caused by several factors. Many people, especially men, prefer not to seek healthcare.^35^ Women are clinically diagnosed with depression far more often than men, probably not only because they are more depressed but also because they are more likely to seek healthcare, thus increasing the chance that their depression will be detected.^36^ Also, diagnostic criteria for depression originate from a female norm and symptoms provided by women, leading to an increased likelihood that depression in women will be diagnosed.^15^ Depression in men has a different presentation than the classic depressive symptoms more often than in women, and this could lead to men's mental health problems not being recognised and therefore being under-treated.^13,35^

In contrast to diagnosed depression, previous studies using HADS to assess depression have mostly found no gender differences or, like our study, found a higher prevalence of depression in men.^37,38^ Since men experience more melancholic symptoms and women more somatic symptoms (e.g. increased appetite and weight, and hypersomnia),^13–17^ the reversed gender differences in depression assessed with HADS compared with clinically diagnosed depression could be due to the fact that HADS's focus is more on melancholic rather than somatic symptoms. Whether the HADS might be more sensitive than other scales in detecting depression in men is as yet unclear.

Other factors that could explain the relatively low use of ADs in our study among the sample with depression might be that the depressed participants may have been reluctant to accept treatment with ADs, a choice that is possibly more common among men; they may not have needed drug treatment, perhaps because other treatments were used (in mild depression psychotherapy is considered as effective as drugs); or they may not have had the prescribed drug dispensed. Many patients do not adhere to treatment instructions, for example do not even obtain their prescribed drugs (primary non-adherence), and prior studies have suggested that both gender and illness severity affect adherence.^39–42^

In our study, it was twice as common for women as for men to use ADs when not currently depressed. This could indicate that their depression was in remission, but it could also mean that women are being over-treated with ADs. Several studies have found AD use to be higher among women, and the increased prescription of ADs in recent decades is especially notable among women.^23–25,30,43^ The higher level of AD prescribing to women may in part be attributed to the greater consumption of healthcare among women in general.^36,44^ Apropos of this, there are studies that show that women are more likely than men to receive a prescription during their medical visits.^45^ The lower threshold for prescribing ADs has led to a debate about the possibility of over-prescription or of ADs being sometimes prescribed where alternatives would be better.^7–9^ It seems that even mild symptoms are now considered indicative of disease and treated with medications, although the efficacy is often limited in mild to moderate depression.^7,46,47^ Further, an expanding number of indications (e.g. neuropathic pain, anxiety disorders, eating disorders and sleep disorders) seen more often in women than in men are contributors to the increasing trend to prescribe ADs, and this could explain some of the AD use without depression seen in our study.^9,48^

As in other studies, the SSRIs were the main drugs in our study.^28,30^ Because of gender differences in the pharmacokinetics and pharmacodynamics of ADs, and because depression may present differently in women and men, it has been suggested that men and women could differ in their response to treatment and that pharmacological treatments should therefore be chosen by gender.^49–51^ The women in our study used SSRIs more often than the men. It could be that somatic symptoms respond better to SSRIs than to TCAs. Another cause might be gender differences in adverse drug reactions. However, supporting data are limited and sometimes conflicting, and current treatment guidelines do not take gender into account.^49,50,52^ Regardless, it is important to continue to examine any differences between men and women concerning pharmacotherapeutic efficacy and adverse drug reactions.

### Limitations

The SPDR offers complete data on all dispensed drugs; however, it does not give information on actual usage. Also, ADs during the 6-month period were analysed without distinguishing whether the drugs had been used for a long or a short time. Moreover, ADs are sometimes prescribed for indications other than depression, which we could not control for. However, previous studies have reported that depression remains the main indication for AD use.^53^ Participation in the study was voluntary and there may have been selection biases. For example, non-responders were more likely to be men than women. It is also possible that people with current symptoms of depression would be less likely to respond, introducing further bias to participant selection. Depression assessed using the HADS (in the previous week) was not directly linked to prescribed ADs via the SPDR (0–6 months prior to the HADS evaluation). However, depression is often a prolonged state, and problems in this respect that were encountered in the previous week were probably not temporary. Finally, it is important to emphasise that a cross-sectional design does not permit evaluation of causality to be derived from the results.

### Summary of findings

The relationship between self-reported depression and use of ADs differed by gender. Overall, men were prescribed ADs to a lesser extent than women, although they reported depression to a greater extent. By contrast, women were prescribed ADs without reporting depression more often than men. This may be a sign for under-treatment among men and over-treatment among women. Further, men and women were prescribed different types of ADs, possibly because of gender differences in treatment outcomes and adverse drug reactions. Although the causes of these findings remain unclear, diagnostic and treatment guidelines should benefit from considering gender in these respects.
